# Supplementary material for: The origin and evolution of open habitats in North America inferred by Bayesian deep learning models
Source: Nat Commun. 2022 Aug 17;13:4833. doi: 10.1038/s41467-022-32300-5 (PMC9385654; doi:10.1038/s41467-022-32300-5)
Supplement: Supplementary file 1 — Supplementary information [file 41467_2022_32300_MOESM1_ESM.pdf]

Supplementary Information for

The origin and evolution of open habitats in North America inferred by deep learning models

Tobias Andermann*, Caroline A.E. Strömberg, Alexandre Antonelli, Daniele Silvestro*

***Corresponding authors. tobias.andermann@ebc.uu.se, daniele.silvestro@unifr.ch**

This file includes:

- Supplementary Discussion
- Supplementary Table 1
- Supplementary Figures 1-10

Other Supplementary Materials for this manuscript include the following:

- Supplementary Data 1 and 2
  (available at https://doi.org/10.5281/zenodo.6492100)

Supplementary Discussion

Evaluating prediction accuracy on current vegetation map

For the task of predicting palaeovegetation, which is presented in the main text, we only have limited data points with past vegetation information (those compiled through the literature review). This limits our ability to evaluate how accurately the trained models predict the true vegetation patterns, based on these few subsampled data points. Yet, the current vegetation data for North America provides a large dataset and thus a suitable framework to test the ability to predict the overall vegetation pattern correctly.

To investigate how well our model can predict vegetation only based on a small subsample of the actual vegetation, we trained a BNN model using only 281 of the approximately 11,000 current vegetation points across North America (Supplementary Fig. 3a and b). This number of training points is equal to the number of palaeovegetation points used for training of the palaeo-models, although in case of the palaeo-data these points are distributed throughout several geological stages. While these training data only constitute about 2.5% of the current vegetation information, our model was able to predict the entire current vegetation (Supplementary Fig. 3c), with a prediction accuracy of 88.1% (Table 1). When making visible where on the map our model misclassified vegetation labels, we find that these misclassifications predominantly occur in the transitioning areas between open and closed vegetation (Supplementary Fig. 3d). This suggests that, while our model accurately learns the general distributions of both vegetation types, the misclassifications are mainly a result of limited resolution of the vegetation boundaries, likely caused by the relatively small number of training vegetation points.

We find that a 5-fold increase in the number of training points (n=1,405) increases the resolution of the predictions, leading to an increased prediction accuracy of 91.6% (Table 1, Supplementary Fig. 4). This shows that more data points can increase the accuracy of the model, suggesting that our model accuracies presented in the main text could be further improved with more palaeovegetation data points being available. However, this bottleneck of limited training data and the resulting limitations in prediction accuracy can be partly accommodated by applying posterior thresholds to the vegetation predictions (see below).

Applying posterior thresholds to increase accuracy

One of the advantages of using BNNs over regular neural network implementations is the explicit modeling of uncertainty in the model predictions by producing posterior probability (PP) estimates for each vegetation prediction (see Methods for more detailed information). These PPs are derived from a posterior sample of the BNN weights and are thus different in their nature to the class-probabilities resulting from the output layer of a regular neural network, which instead represent point estimates. We utilize the PP estimates by setting a posterior threshold to only make vegetation predictions for sites that have a high prediction certainty, while assigning all predictions below the thresholds as “unknown”. We select this PP threshold individually for each model to ensure a specified minimum prediction accuracy.

In the case of our model trained on 281 current vegetation points, we find that a PP threshold of 0.56 leads to an expected prediction accuracy of > 90% (Table 1), while still allowing to make vegetation predictions for 94.5% of all North American terrestrial cells (Supplementary Fig. 5). Increasing the PP threshold to 0.74 leads to an expected prediction accuracy of > 95%, while predicting 77.2% of the map with high confidence (Supplementary Fig. 3e). For the model trained on 1,405 current vegetation labels, a posterior threshold of 0.66 was sufficient to ensure 95% prediction accuracy, allowing to make high accuracy vegetation predictions for 89.5% of North American terrestrial cells (Supplementary Fig. 4). Prediction accuracies even higher than 95% could be achieved by setting higher posterior thresholds, at the cost of an increasing number of predictions labeled as unknown (Supplementary Fig. 5). This trade-off between quality and quantity of the predictions allows us to focus the predictions of the model only on areas for which we can confidently infer vegetation, leading to a substantial increase in prediction accuracy (Supplementary Fig. 3f). This property of BNN models is of particular interest when the number of available vegetation information for training is limited, as is the case for our palaeovegetation models.

Supplementary Tables

Supplementary Table 1. Association scores of mammal and plant taxa with open habitat. The openness score was calculated as the fraction of occurrences of each taxon that fall within predicted open habitat, averaged across the predicted 1 Ma increments throughout the last 30 Ma.

Taxon	Openness score	Taxon	Openness score	Taxon	Openness score
Cratogeomys	1.00	Sorex	0.59	Lauraceae	0.27
Onychomys	1.00	Lynx	0.57	Procyon	0.26
Baiomys	1.00	Gomphotherium	0.57	Tamias	0.26
Tayassu	1.00	Ammospermophilus	0.56	Myricaceae	0.21
Notiosorex	1.00	Lepus	0.56	Fabaceae	0.21
Dipodomys	1.00	Adoxaceae	0.55	Mammut	0.19
Cynomys	0.98	Ondatra	0.54	Odocoileus	0.18
Brachyerix	0.87	Platygonus	0.54	Vitaceae	0.17
Reithrodontomys	0.82	Cryptotis	0.53	Typhaceae	0.16
Perognathus	0.82	Ursus	0.52	Dasypus	0.16
Taxidea	0.80	Panthera	0.51	Oleaceae	0.16
Eumops	0.80	Oryzomys	0.51	Ulmaceae	0.16
Equus	0.78	Blarina	0.50	Juglandaceae	0.15
Dipoides	0.78	Mustela	0.49	Berberidaceae	0.15
Spermophilus	0.76	Marmota	0.49	Sciurus	0.15
Antrozous	0.74	Myotis	0.48	Asteraceae	0.14
Geomys	0.73	Phenacomys	0.48	Rhamnaceaee	0.14
Ochotona	0.73	Scapanus	0.48	Platanaceae	0.14
Thomomys	0.72	Meliaceae	0.47	Cyperaceae	0.14
Puma	0.72	Anacardiaceae	0.47	Polygonaceae	0.13
Spilogale	0.71	Castor	0.46	Salicaceae	0.13
Bassariscus	0.70	Mammuthus	0.46	Sapindaceae	0.11
Aphelops	0.70	Tapirus	0.45	Fagaceae	0.11
Canis	0.69	Boraginaceae	0.44	Rosaceae	0.11
Neotoma	0.69	Eptesicus	0.43	Ericaceae	0.11
Lasionycteris	0.67	Peromyscus	0.42	Grossulariaceae	0.09
Hypolagus	0.63	Felis	0.41	Betulaceae	0.09
Poaceae	0.63	Vulpes	0.41	Caprifoliaceae	0.05
Cannabaceae	0.63	Ranunculaceae	0.39	Cornaceae	0.02
Sigmodon	0.62	Ebenaceae	0.39	Potamogetonaceae	0.02
Mephitis	0.60	Lontra	0.36	Nymphaeaceae	0.02
Zapus	0.60	Erethizon	0.36	Altingiaceae	0.02
Urocyon	0.59	Scalopus	0.32		
Sylvilagus	0.59	Hydrangeaceae	0.28		

Supplementary Figures

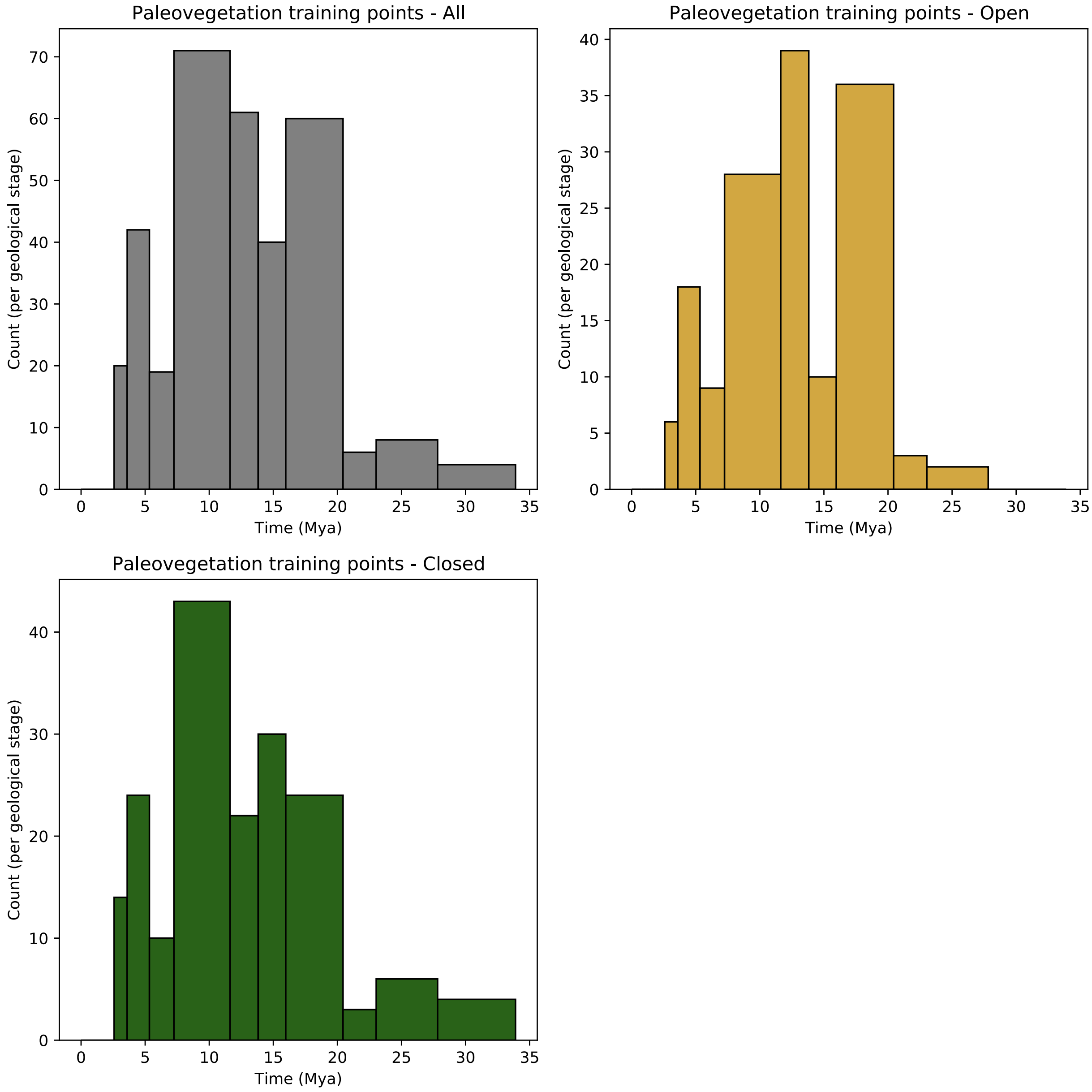

Supplementary Figure 1. Temporal distribution of palaeovegetation sites analyzed. The ages shown in the histograms were binned using the geological stage boundaries according to the International Chronostratigraphic Chart, v2020/03. The first histogram (grey) shows all palaeovegetation points used in this study for training. The second plot (yellow) shows only the open vegetation points, and the third plot (green) shows only the closed vegetation points.

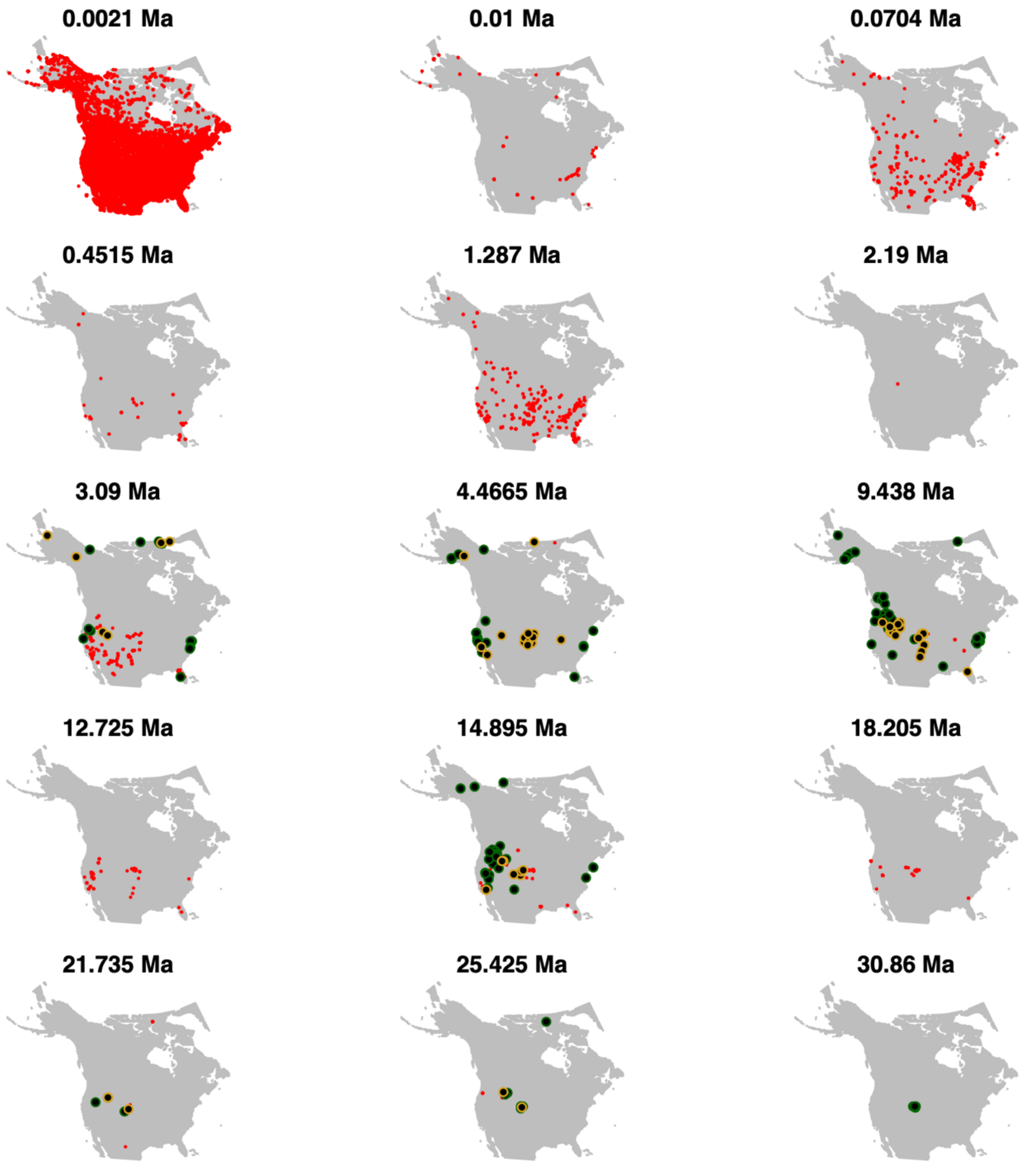

Supplementary Figure 2. Spatial and temporal distribution of taxon occurrence data and palaeovegetation data used in this study, plotted for each geological stage. All spatial data are transformed in Albers equal area projection. Red dots show occurrence points of taxa (current or fossil occurrences), green points show closed vegetation data points, and yellow points show open vegetation data points. Note that distances for input in our BNN are calculated between a given vegetation point and all taxon occurrences in each geological stage, providing pairs of measures of geographic and temporal distances.

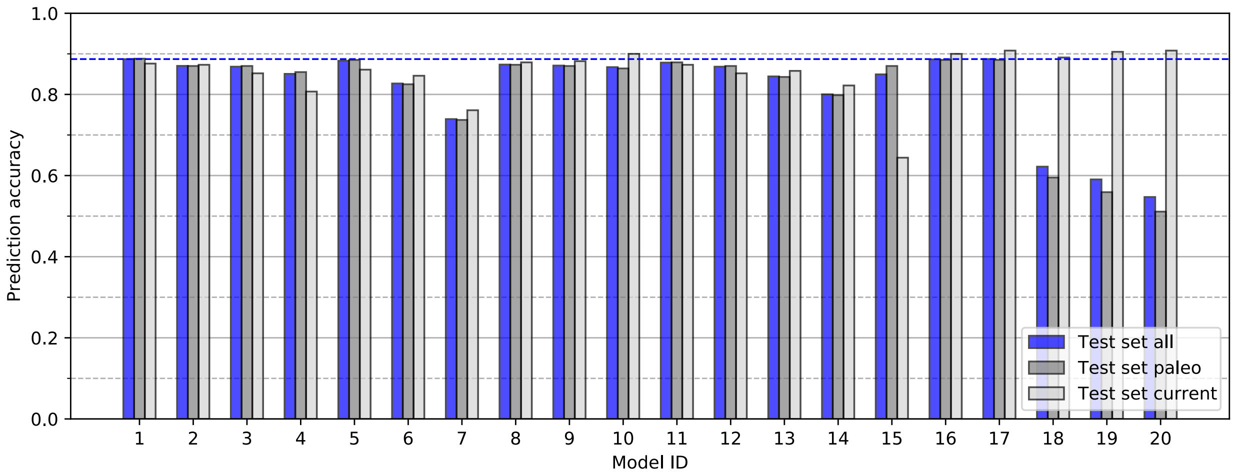

Supplementary Figure 3. Prediction accuracy of tested models. The tested scenarios differed in the number of nodes (32-8 or 8), the used features (all, only biotic, or only abiotic), the pooling strategy applied to the biological features (none, sum-pooling, or max-pooling), and the number of current vegetation instances used for training (0, 331, 662, or 1655, see Table 1 in main text for model ID key). Blue bars show the overall prediction accuracy of each model (five-fold cross validation), which constitutes the weighted mean of the palaeovegetation prediction accuracy (dark grey) and the current vegetation prediction accuracy (light grey. The blue horizontal bar shows the maximum reached prediction accuracy of 88.7% (model 1).

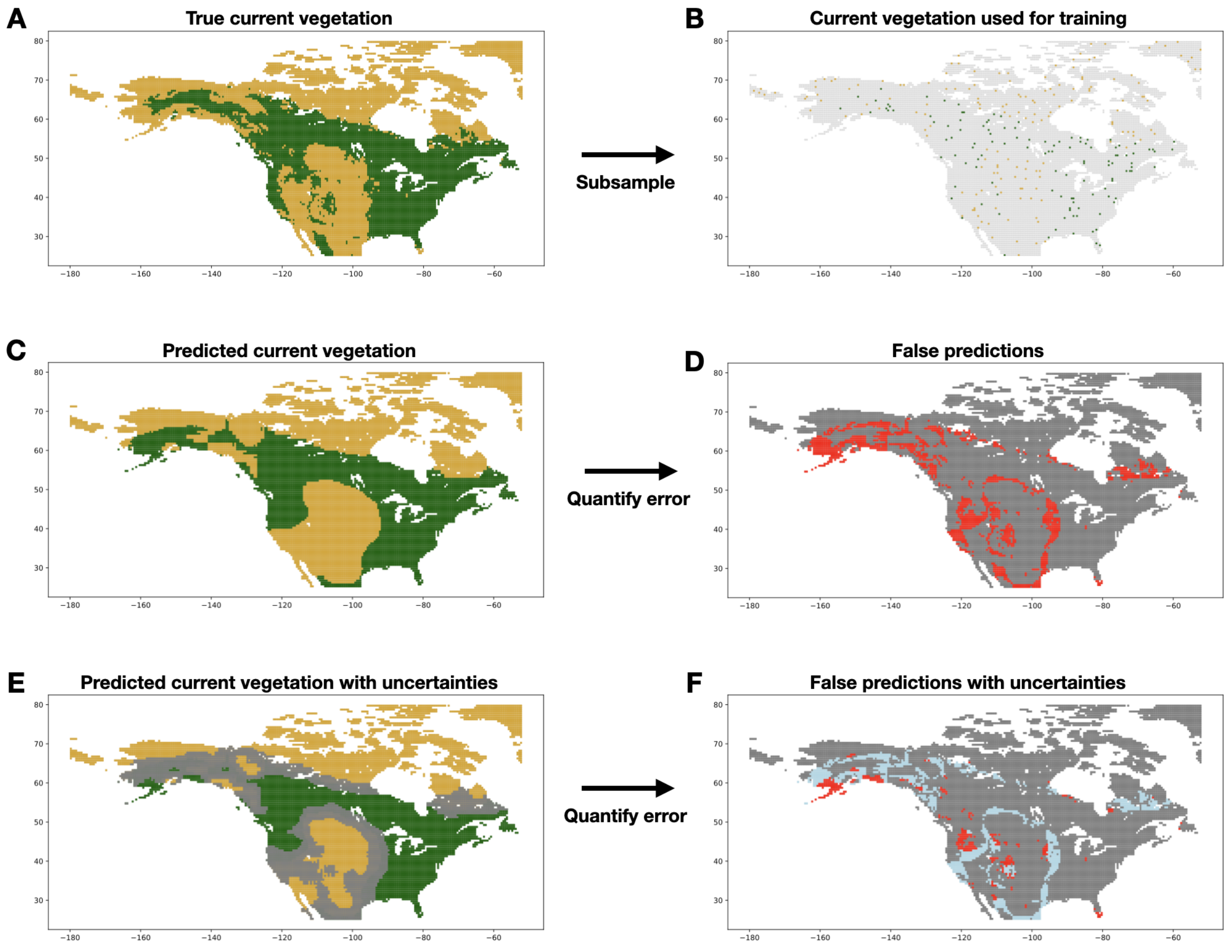

Supplementary Figure 4. Utility of applying posterior probability thresholds to vegetation predictions. The true current distribution of open (yellow) and closed habitat (green) in North America (A) is based on the SYNMAP potential vegetation data. A random subsample containing 281 of these current vegetation points (B) was used for training the BNN model. We used the trained model to predict the current vegetation of North America, based on the mammal, plant, and climate associations with the vegetation type that were learned by the model (C). While the majority of the current map was predicted correctly (84.8% accuracy, excl. training points), there are several incorrect vegetation predictions, highlighted in red (D). A posterior threshold can be applied to our model predictions (E), which allows us to distinguish between confident vegetation predictions (colored), and those the model is uncertain about (grey). In this case we applied a posterior threshold that ensures a prediction accuracy of > 95%. The resulting predictions show only a small fraction of falsely predicted vegetation labels (F), while the majority of the problematic predictions are now modeled as uncertain (light blue).

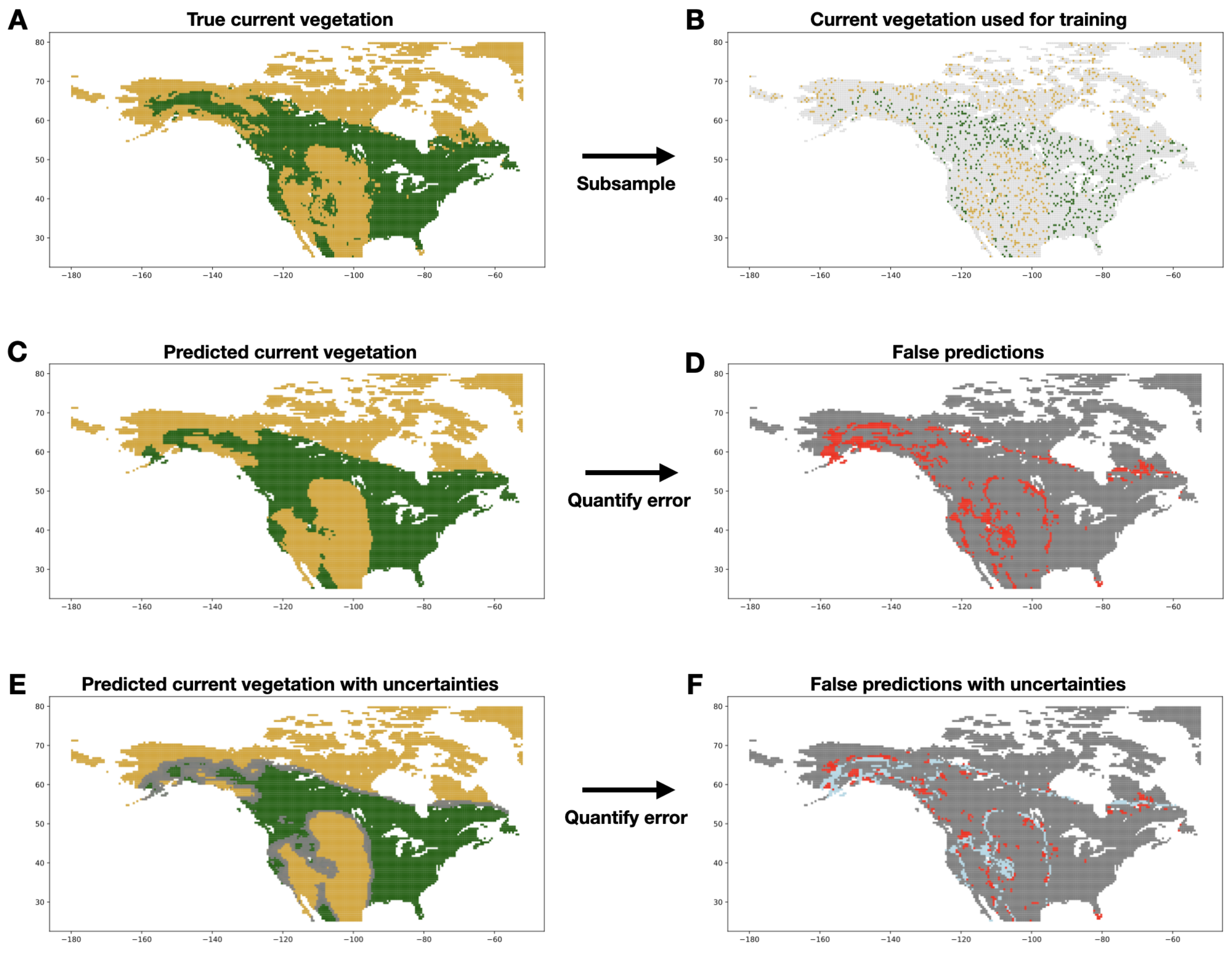

Supplementary Figure 5. Predictions of a model trained with 1,405 current vegetation sites. (A) The true current distribution of open (yellow) and closed habitat (green) in North America; (B) a random subsample containing 1,405 of these current vegetation points which were used for training the model; (C) predictions of current vegetation made by the trained model; (D) incorrect vegetation predictions, highlighted in red; (E) vegetation predictions after applying posterior threshold, with uncertain predictions colored in grey; (F) after applying the posterior threshold, the majority of incorrect predictions are modeled as uncertain (light blue), with remaining false predictions colored in red. See caption of Supplementary Fig. 3 for more details.

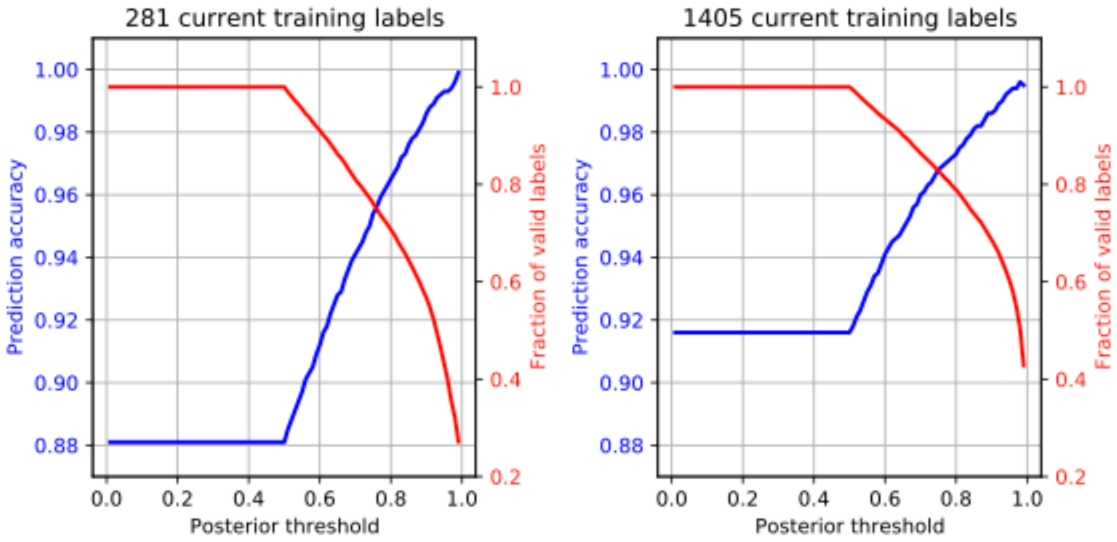

Supplementary Figure 6. Trade-off between increasing prediction accuracy and fewer vegetation label predictions with increasingly strict posterior thresholds. Results are shown for a model trained on 281 current vegetation labels (no palaeontological data), as well as for a model with a 5-fold increased number of current training labels (n=1,405). The displayed accuracies were calculated based on all current vegetation labels across North America, excluding the labels used for training.

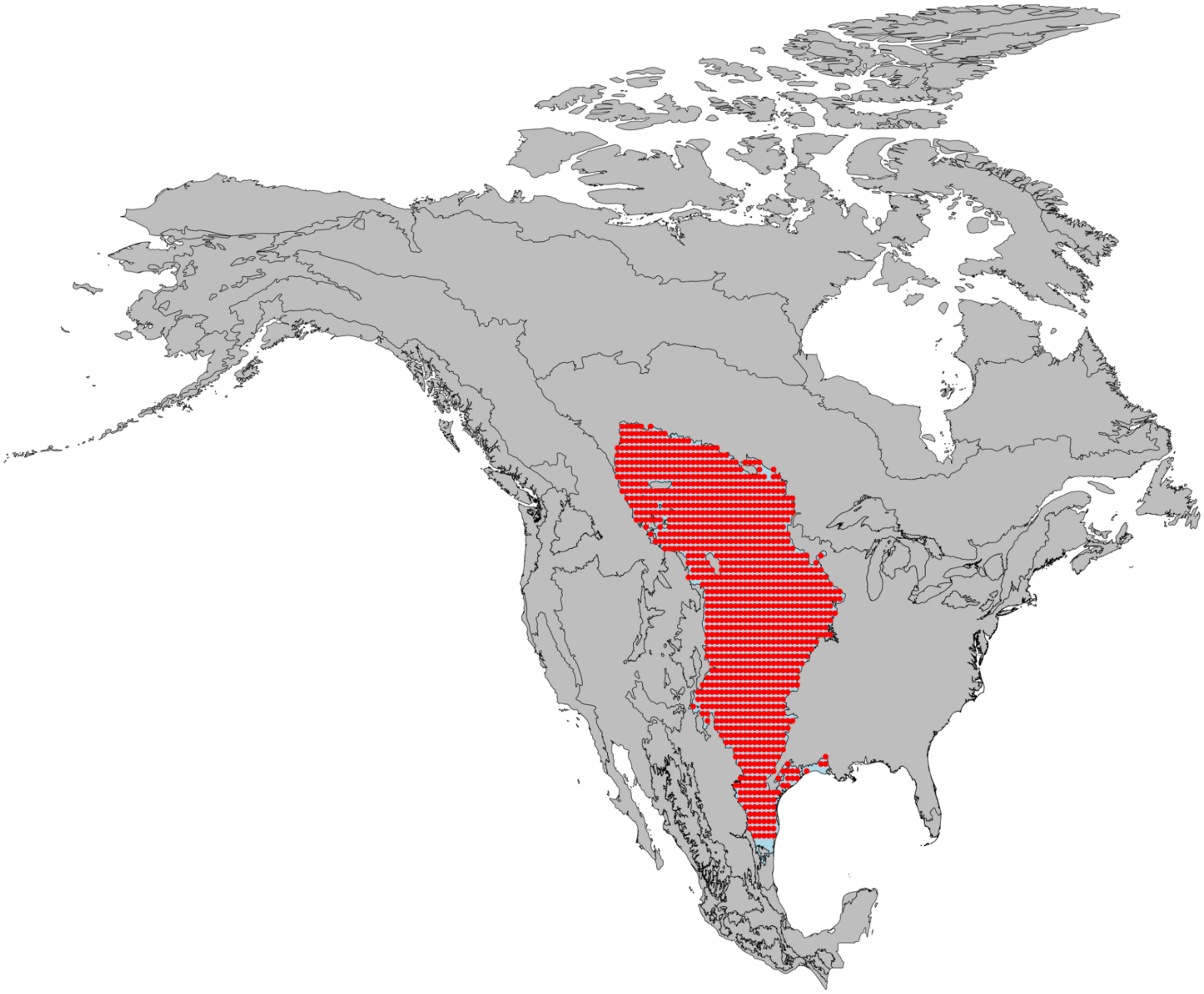

Supplementary Figure 7. The American Great Plains ecoregion, as defined by Omernik et al., 2014 (ref. 29 in main text). The polygon for this ecoregion (light blue) was downloaded from https://www.epa.gov/eco-research/ecoregions-north-america (Level 1 ecoregion), and was used in this study to select all grid cells (red dots) that fall within this ecoregion. For these grid cells we compiled the vegetation predictions through time separately to observe the dynamics of open vegetation expansion for a more spatially explicit region.

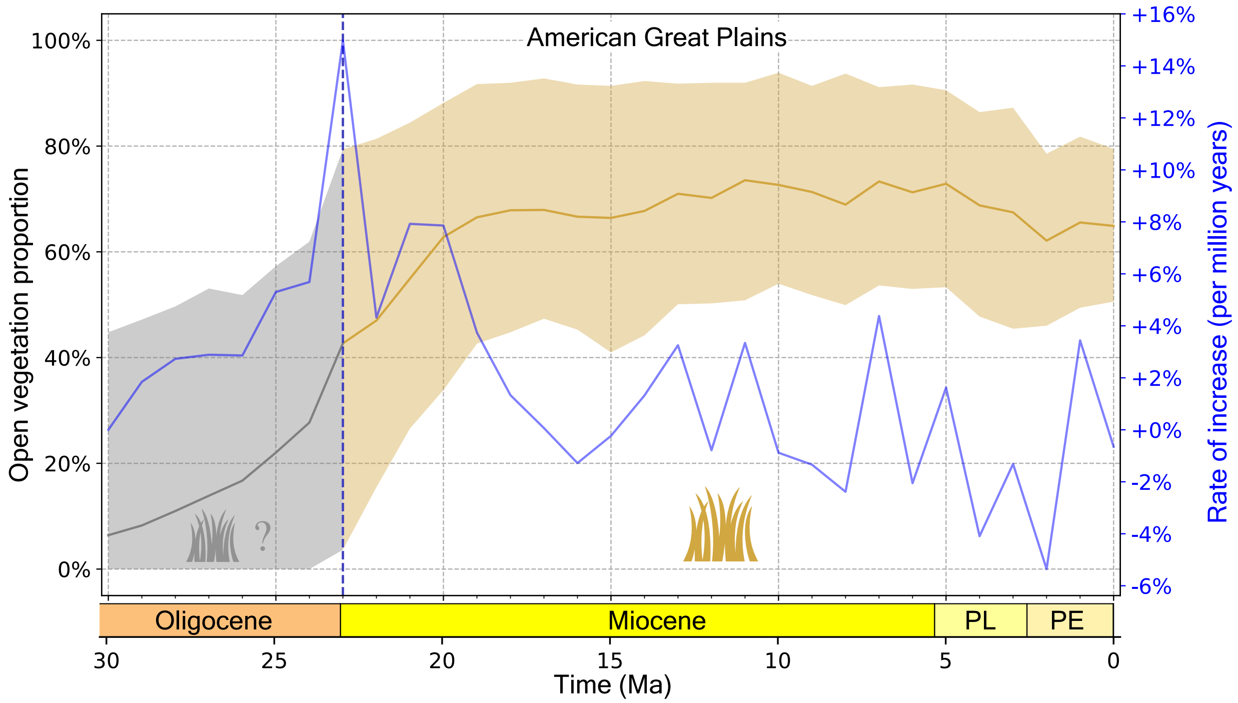

Supplementary Figure 8. Proportion of predicted open habitat through time, compiled for the Great Plains ecoregion. The solid yellow line shows the mean posterior estimates, while the shaded area shows the 95% HPD interval. The blue line shows the mean rate of open habitat expansion over the preceding 1-million-year time bin. The x-axis shows time in million years, the y-axis to the left shows the percentage of open habitat, while the y-axis to the right (blue) shows the rate of open vegetation increase in percent (per million years).

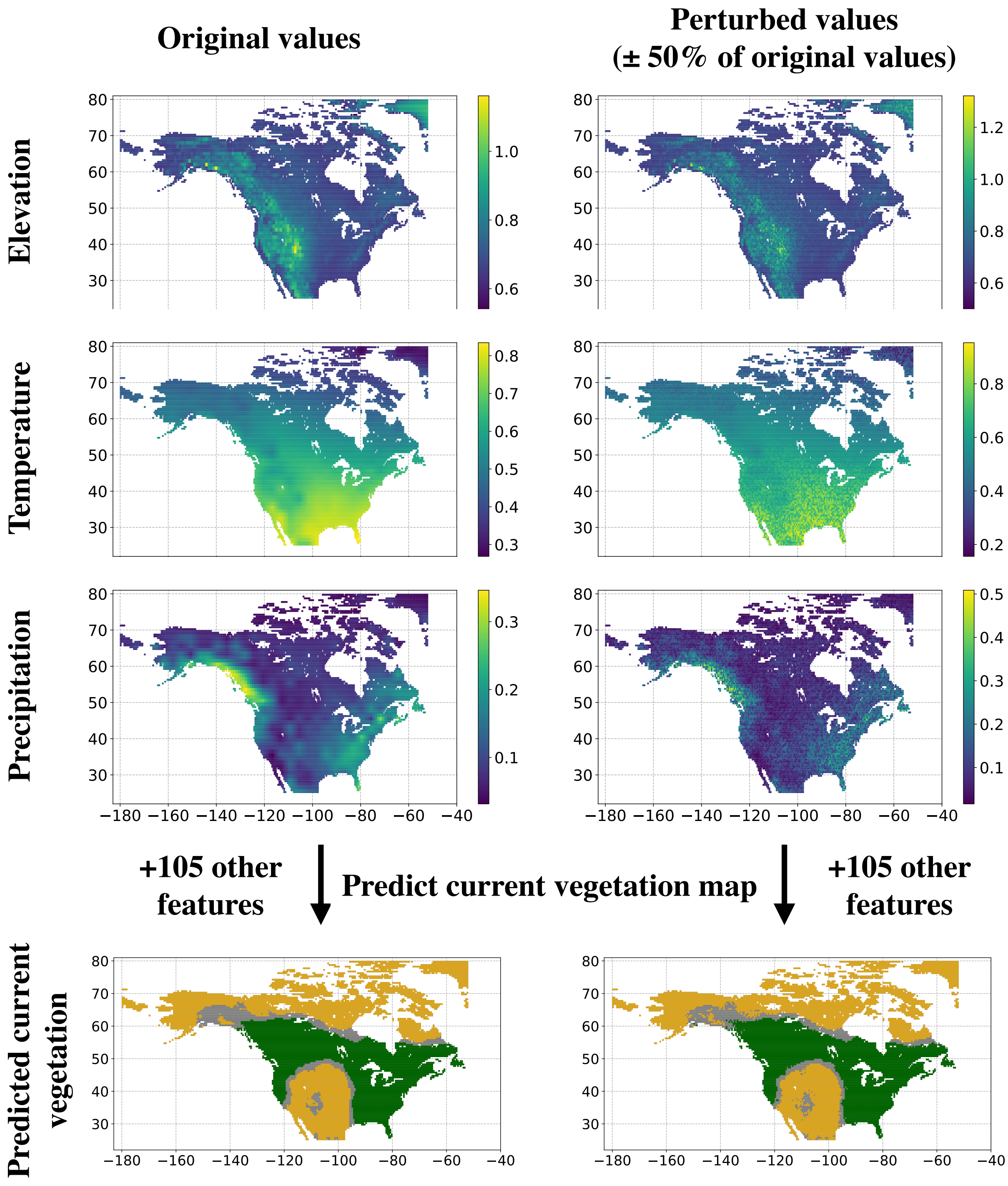

Supplementary Figure 9. The effect of perturbating climatic and elevation features. Since the grid data used as predictors for elevation, temperature, and precipitation are based on modeled values, we tested how robust our model predictions are when randomly perturbing these predictor values. We find that perturbing these data does not affect the produced vegetation patterns (see bottom panels).

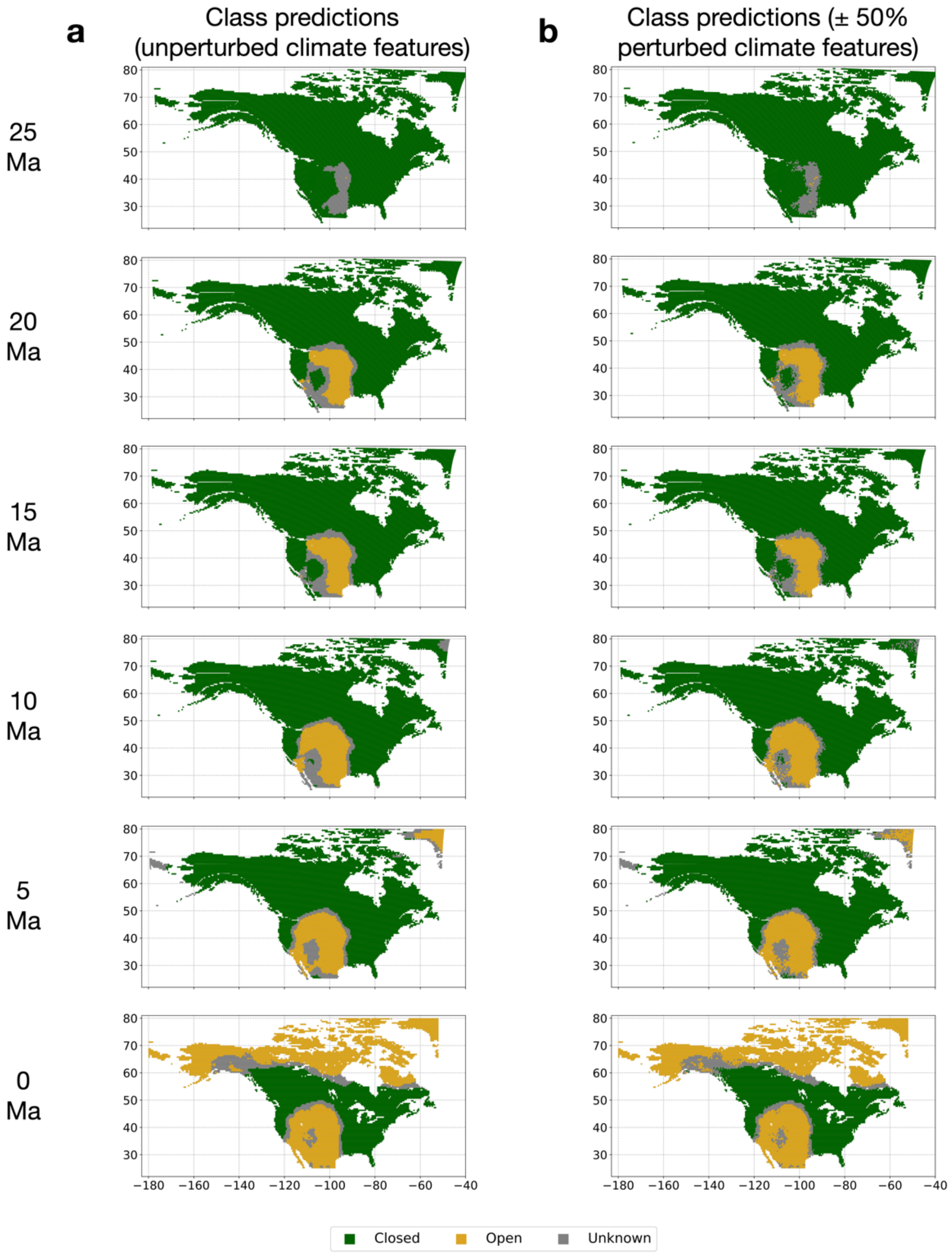

Supplementary Figure 10. Vegetation predictions through time compared between the original feature data (a) and the modified feature data with randomly perturbed values for the modeled elevation, temperature, and precipitation feature data (b). The predicted pattern for open vegetation expansion through time remains consistent, despite the data for these three features being perturbed by ± 50% around the original value.
