# Supplementary material for: The origin and evolution of open habitats in North America inferred by Bayesian deep learning models
Source: Nat Commun. 2022 Aug 17;13:4833. doi: 10.1038/s41467-022-32300-5 (PMC9385654; doi:10.1038/s41467-022-32300-5)
Supplement: Supplementary file 3 — Description of Additional Supplementary Files [file 41467_2022_32300_MOESM3_ESM.pdf]

Description of Additional Supplementary Files

File Name: Supplementary Data 1
Description: List of sites with compiled paleovegetation information.

File Name: Supplementary Data 2
Description: List of all fossil occurrences used in this study.
