# Supplementary material for: The origin and evolution of open habitats in North America inferred by Bayesian deep learning models
Source: Nat Commun. 2022 Aug 17;13:4833. doi: 10.1038/s41467-022-32300-5 (PMC9385654; doi:10.1038/s41467-022-32300-5)
Supplement: Supplementary file 6 — Reporting Summary [file 41467_2022_32300_MOESM6_ESM.pdf]

nature portfolio

Corresponding author(s): Tobias Andermann

Last updated by author(s): Jun 1, 2022

Reporting Summary

Nature Portfolio wishes to improve the reproducibility of the work that we publish. This form provides structure for consistency and transparency in reporting. For further information on Nature Portfolio policies, see our Editorial Policies and the Editorial Policy Checklist.

Statistics

For all statistical analyses, confirm that the following items are present in the figure legend, table legend, main text, or Methods section.

n/a	Confirmed	
☐	☒	The exact sample size (*n*) for each experimental group/condition, given as a discrete number and unit of measurement
☐	☒	A statement on whether measurements were taken from distinct samples or whether the same sample was measured repeatedly
☐	☒	The statistical test(s) used AND whether they are one- or two-sided<br>*Only common tests should be described solely by name; describe more complex techniques in the Methods section.*
☐	☒	A description of all covariates tested
☐	☒	A description of any assumptions or corrections, such as tests of normality and adjustment for multiple comparisons
☐	☒	A full description of the statistical parameters including central tendency (e.g. means) or other basic estimates (e.g. regression coefficient) AND variation (e.g. standard deviation) or associated estimates of uncertainty (e.g. confidence intervals)
☐	☒	For null hypothesis testing, the test statistic (e.g. *F*, *t*, *r*) with confidence intervals, effect sizes, degrees of freedom and *P* value noted<br>*Give P values as exact values whenever suitable.*
☐	☒	For Bayesian analysis, information on the choice of priors and Markov chain Monte Carlo settings
☐	☒	For hierarchical and complex designs, identification of the appropriate level for tests and full reporting of outcomes
☒	☐	Estimates of effect sizes (e.g. Cohen's *d*, Pearson's *r*), indicating how they were calculated

Our web collection on statistics for biologists contains articles on many of the points above.

Software and code

Policy information about availability of computer code

Data collection	All scripts that were used to download and process the data are available on the project's GitHub repository https://github.com/tandermann/paleovegetation (https://doi.org/10.5281/zenodo.6604119).
Data analysis	All scripts that were used to analyze and plot the data are available on the project's GitHub repository https://github.com/tandermann/paleovegetation (https://doi.org/10.5281/zenodo.6604119). Additionally, we used functions available in the BNN python library at https://github.com/dsilvestro/npBNN, v0.1.12. We used the mapast R-package (v0.1) to track continental movement. We used a function of the program PyRate (v3.0) to correct misspellings of taxon names in the fossil data.

For manuscripts utilizing custom algorithms or software that are central to the research but not yet described in published literature, software must be made available to editors and reviewers. We strongly encourage code deposition in a community repository (e.g. GitHub). See the Nature Portfolio guidelines for submitting code & software for further information.

Data

Policy information about availability of data

All manuscripts must include a data availability statement. This statement should provide the following information, where applicable:

- Accession codes, unique identifiers, or web links for publicly available datasets
- A description of any restrictions on data availability
- For clinical datasets or third party data, please ensure that the statement adheres to our policy

The supplementary material accompanying this manuscript contains Supplementary Discussion, Supplementary Figures 1-10, and Supplementary Table 1. Additionally, Supplementary Data 1 and 2 are available in the Zenodo repository https://doi.org/10.5281/zenodo.6492100. The repository also contains all datasets analyzed and generated during the current study, as well as source data for all figures and tables.

Data sources used in this study:
- Paleovegetation reconstructions from peer-reviewed literature (see Supplementary Data 1)
- Current vegetation information from SYNMAP Global Potential Vegetation data (https://databasin.org/datasets/112a942ec4294e5284e63d5e6bf14b29/)
- Mammal fossil data from Paleobiology Database (https://paleobiodb.org/, see Supplementary Data 2)
- Plant fossil data from Cenozoic Angiosperm database (https://doi.org/10.1086/685388, see Supplementary Data 2)
- Current taxon occurrences from GBIF (download DOI: https://doi.org/10.15468/dl.nxuyg8)
- Elevation rasters through time (https://zenodo.org/record/5460860)
- Paleotemperature and paleovegetation data through time (https://doi.org/10.1146/annurev-earth-081320-064052). Restrictions apply to the availability of these data, which were used under license for the current study, and so are not publicly available. Data are however available from the authors upon reasonable request and with permission of Christopher Scotese (cscotese@gmail.com).

Field-specific reporting

Please select the one below that is the best fit for your research. If you are not sure, read the appropriate sections before making your selection.

☐ Life sciences ☐ Behavioural & social sciences ☒ Ecological, evolutionary & environmental sciences

For a reference copy of the document with all sections, see nature.com/documents/nr-reporting-summary-flat.pdf

Ecological, evolutionary & environmental sciences study design

All studies must disclose on these points even when the disclosure is negative.

Study description	Here we use publicly available data (fossil occurrences, climatic predictors, past vegetation reconstructions form individual sites) to train machine learning models for the task of vegetation inference. We demonstrate how the trained models can be used to predict vegetation through (deep) time and space.
Research sample	All data was restricted to North America (as defined in our study). This selection was done because the paleovegetation data that could be compiled was very limited for other areas, but had a good spatial and temporal spread across the North American continent. For this study we selected all mammal genera with available fossil occurrences (Paleobiology Database, see Data section above) spanning at least half of the time frame of our study (last 30 million years). This resulted in 65 mammal taxa. Additionally we added fossil data of 35 plant families (Cenozoic Angiosperm database), using the same criteria. This was supplemented by all current occurrences available on GBIF for these taxa (https://doi.org/10.15468/dl.nxuyg8).
Sampling strategy	We used all available data that passed our quality filters. Given that we were working on paleodata, the sampling of past epochs was limited by the data availability.
Data collection	Data for this study was collected from public databases and compiled from the peer-reviewed literature. This included current and fossil occurrences of mammals and plants (compiled by Tobias Andermann), as well as reconstructions of past vegetation from individual sites, including their annotation as "open" or "closed" (compiled by Caroline Stromberg). We used Google Scholar to search for relevant publications, as well as the databases listed under the Data section above.
Timing and spatial scale	Temporal scale: from present until 30 million years ago, extracting all available records for each datatype (fossil occurrences and paleovegetation reconstructions, see Supplementary Fig. 2); spatial scale: North America, delimited by a cropping window with corner points P1 (Lon = -180, Lat = 25) and P2 (Lon = -52, Lat = 80).
Data exclusions	No exclusions.
Reproducibility	The scripts provided in the GitHub project repository can be used to reproduce our workflow from the raw data to the final analyses and plots.
Randomization	When training our models, a random selection of paleovegetation points and current vegetation points was selected as training set to train the models and as test set to evaluate the models. To account for the stochasticity and to effectively use all our data, we used the approach of cross validation, using a different subset of the data for training and evaluation and averaging across all trained and evaluated models.
Blinding	No blinding was applied, since all results are entirely data-driven and therefore not affected by observer or confirmation biases.

Did the study involve field work? ☐ Yes ☒ No

Reporting for specific materials, systems and methods

We require information from authors about some types of materials, experimental systems and methods used in many studies. Here, indicate whether each material, system or method listed is relevant to your study. If you are not sure if a list item applies to your research, read the appropriate section before selecting a response.

Materials & experimental systems

n/a	Involved in the study
☒	☐ Antibodies
☒	☐ Eukaryotic cell lines
☒	☐ Palaeontology and archaeology
☒	☐ Animals and other organisms
☒	☐ Human research participants
☒	☐ Clinical data
☒	☐ Dual use research of concern

Methods

n/a	Involved in the study
☒	☐ ChIP-seq
☒	☐ Flow cytometry
☒	☐ MRI-based neuroimaging
